# Nef is a key player in neuroinflammation and myelin impairment associated with neuroHIV

**DOI:** 10.3389/fneur.2025.1553594

**Published:** 2025-03-12

**Authors:** Michael Ilya Bukrinsky

**Affiliations:** School of Medicine and Health Sciences, The George Washington University, Washington, DC, United States

**Keywords:** human immunodeficiency virus (HIV), Nef + exosomes, myelin alteration, inflammation, neuroHIV

## Introduction

Anti-retroviral treatment (ART) has transformed HIV infection into a manageable chronic disease. It restores immune functionality and eliminates or reduces many AIDS-defining co-morbidities. In particular, the severity of HIV-associated neurocognitive disorders (HAND) has been greatly reduced, significantly decreasing the prevalence of HIV-associated dementia (1). However, the overall prevalence of milder forms of HAND remains comparable to the pre-ART era (2–4). Neurocognitive impairment affects nearly 50% of people living with HIV (PLWH), yet the HIV-specific factors responsible for this co-morbidity remain poorly understood. A role for viral proteins such as gp120, Tat, and Nef has been suggested (5–9). Two recent articles have provided evidence that Nef is the key HIV protein responsible for neuroinflammation, myelin impairment, and neuronal injury.

## Summary of studies

Both studies underscore the detrimental effects of Nef on the CNS, particularly in the context of HIV-1 infection. The first study (10) demonstrates the Nef's key role in neuroinflammation and myelin damage in the context of mouse brain infection with EcoHIV, a hybrid virus carrying HIV core and envelope of murine leukemia virus (11). This model is consistent with ART-suppressed HIV infection in people, as EcoHIV establishes latent infection of the murine brain (12). Considering brain inflammation, expression of inflammatory cytokines in mice infected with Nef-deficient EcoHIV was between the levels observed in mock-infected mice and mice infected with Nef-positive EcoHIV, and significantly differed from both (10), indicating that other HIV factors besides Nef contributed to neuroinflammation. This conclusion is in line with proposed role of Tat in neuroinflammation (8, 9). Interestingly, the effects of Nef and the other HIV factors (presumably Tat) on neuroinflammation appear additive, suggesting that they may work throw different mechanisms. Indeed, Tat was suggested to disrupt the BBB and activate NF-kB on monocytes and microglia, promoting migration of activated cells into the brain and production of inflammatory cytokines (13, 14), whereas Nef induces inflammation in myeloid cells by affecting cholesterol homeostasis and lipid rafts (15).

While Nef appears to cooperate with other HIV factors to promote inflammation, the observed effects on myelin impairment and neuronal injury in this model were attributed exclusively to Nef, as no such defects were detected in mice infected with Nef-deficient EcoHIV (10). However, this conclusion may be influenced by the relatively short post-infection observation period in this study (2–3 weeks), as chronic inflammation is expected to contribute to neuronal damage over time. Consequently, the potential contribution of other HIV proteins, such as Tat, to neurotoxicity via the induction of neuroinflammation may have been overlooked. These findings also argue against the direct acute cytotoxicity of Tat proposed in previous studies (16). This discrepancy may arise because the Tat concentrations required to induce cytotoxicity *in vitro* [~400 nM (17)] are likely not achieved in the EcoHIV model, although Tat concentrations in the brain have not been directly measured in this context. Notably, Tat concentrations in the cerebrospinal fluid (CSF) of ART-treated PLWH range from 0.5 to 6.5 ng/mL (36–465 pM) (18), which is significantly lower than the neurotoxic concentrations used in *in vitro* studies. Collectively, these results suggest that Nef is a primary driver of acute neuroinflammation, myelin impairment and neuronal damage in the EcoHIV model of HAND. Further studies are needed to explore the potential long-term contributions of other HIV proteins, such as Tat, to neuropathogenesis in this model.

The second study (19) provides a more in-depth analysis of Nef-mediated brain damage and introduces the concept of Nef-containing EVs as key mediators of myelin impairment and oligodendrocyte injury. Instead of injecting HIV directly, in this study mice were injected in the brain with Nef EVs, a strategy informed by growing evidence of EVs' roles in neurological diseases (20). Nef is efficiently incorporated into EVs, and these vesicles have been detected in the blood of PLWH with undetectable HIV loads (21). Since EVs can routinely cross the blood-brain barrier (22), they may serve as a vehicle for Nef to reach and affect the central nervous system. Furthermore, Nef has been identified in the brains of ART-treated PLWH (23) and SIV-infected monkeys (24), and it can be taken up by neighboring cells, including neurons, leading to neuronal damage (25). This mechanism is particularly significant because it offers a novel explanation for how Nef may propagate neurotoxic effects throughout the brain without requiring direct viral infection of every affected cell. Importantly, even when HIV replication is suppressed to undetectable levels by ART, Nef EVs continue to be produced (26) and remain capable of exerting neurotoxic effects.

This study demonstrated that Nef-containing EVs disrupted myelin sheaths and inflicted damage upon glial cells, in particular oligodendrocytes, within the murine CNS. The damage to oligodendrocytes was partially prevented by agents that blocked Nef-mediated inhibition of the activity of cellular cholesterol transporter, ABCA1, suggesting that the effects of Nef EVs on myelination were mediated by alterations in cholesterol homeostasis, a known feature of Nef EVs (15). In addition, Nef EVs promoted inflammatory responses by significantly increasing the number of activated microglial cells at the sites of injection.

Both studies identified similar neurotoxic effects of Nef, including pro-inflammatory activity and myelin impairment. Since Nef EVs were not detected in the EcoHIV study, it remains unclear whether the observed effects were driven solely by EcoHIV infection of microglial cells or by a combination of direct infection and EV-mediated toxicity. A more likely scenario is that both mechanisms contribute, with infected microglia triggering inflammation while Nef EVs induce demyelination. This is supported by the low levels of myelin impairment observed in that study (10), which are consistent with Nef EV levels falling below the limit of detection.

When related to HIV infection and HAND, the findings from these two studies support the following model: Under ART treatment, HIV persists in brain-resident cells, particularly microglia and astrocytes, which produce Nef EVs. Additionally, Nef EVs originating from peripheral sources enter the brain via the bloodstream. HIV-infected microglia adopt a pro-inflammatory phenotype, releasing inflammatory cytokines, while Nef EVs further exacerbate inflammation and disrupt myelin integrity. Together, these effects contribute to neuronal damage and impaired synaptic communication, ultimately leading to cognitive deficits.

The two studies reviewed here have several limitations. They did not specifically assess Nef's direct neurotoxicity, though prior research suggests Nef may induce neuronal injury via caspase activation and free radical production (27). However, neuronal death is not a defining feature of the mild forms of HAND that are most prevalent in the ART era (28). Beyond its role in neuroinflammation and myelin impairment, Nef may also disrupt the blood-brain barrier (29), potentially exacerbating the pathogenic mechanisms discussed in this review. Additionally, Nef-driven inflammation could be amplified by its stimulation of CCL5 in microglia (30), creating a feedback loop that sustains neuroinflammatory damage. These aspects, along with the role of Nef EVs in the EcoHIV model, remain unexamined.

Clarifying Nef's role in HAND pathogenesis not only deepens our understanding of the disease but also identifies promising therapeutic targets for preventing neurocognitive decline in PLWH.

## Relevance to HAND in PLWH

The relevance of the EcoHIV model to HAND in PLWH remains incompletely understood. A key limitation of this model is the absence of gp120, a viral protein implicated in neuropathogenesis, which restricts investigations into its role and likely reduces the overall pathological impact. Additionally, unlike HIV-1, which enters cells via CD4/CCR5 or CD4/CXCR4, EcoHIV utilizes mCAT-1, a receptor broadly expressed across various mouse tissues, including the brain. Despite this difference, EcoHIV primarily infects CD4+ T cells and monocytes/macrophages in the periphery (11), while in the brain, it predominantly resides in myeloid cells (31, 32).

Notably, EcoHIV-infected mice develop neurocognitive impairment (NCI) resembling that seen in ART-treated HIV-infected individuals (32, 33). In these mice, infected microglia play a central role in NCI pathogenesis, mirroring findings in HIV-infected humans (34). Specifically, hippocampus- and amygdala-dependent deficits in memory consolidation and recall observed in EcoHIV-infected mice (32) closely parallel cognitive impairments in ART-treated PLWH (35). Additionally, the selective loss of dopaminergic neurons—without significant damage to non-dopaminergic neurons—in the substantia nigra and subventricular zones of EcoHIV-infected mice (36) mirrors aspects of HIV-associated neurotoxicity (37). Crucially, NCI in EcoHIV-infected mice depends on the persistent replication of the virus within brain microglia, which continues despite immune responses and ART (32), resembling the persistence of HIV in the brains of PLWH with mild forms of HAND (38). While differences exist between HIV infection in the human brain and EcoHIV infection in the mouse brain, the resulting neurological impairments share striking similarities. Given these parallels, it is reasonable to infer that the underlying mechanisms of neuropathology in both infections are also closely related.

Neuroinflammation is a hallmark of HAND and is widely recognized as a primary driver of neuronal injury and cognitive decline (39–42). The pro-inflammatory effects of Nef on human myeloid cells (15, 43, 44), along with its presence in post-mortem brain tissues of individuals (23) and monkeys (24) with HAND, highlight its potential role in driving neuroinflammation.

Beyond Nef, other HIV proteins, including Tat and gp120, have also been shown to exert pro-inflammatory effects (45, 46). Additionally, systemic inflammation resulting from the leakage of bacterial products through the gut due to incomplete restoration of the gut mucosa (“leaky gut”) has been implicated as a contributing factor (47). The relative contributions of these factors to neuroinflammation in PLWH remain unclear and require further investigation.

Less is known about Nef's role in myelin impairment. However, myelin loss and disruption are consistently observed in the brains of individuals with HAND (28, 48) and SIV-infected monkeys (49, 50). Notably, Nef has been detected in brain regions critical for cognition and motor function, where demyelination is evident (48). Together with previous findings demonstrating Nef's pathogenic effects on oligodendrocytes (51) and the attenuation of these effects in monkeys infected with Nef-attenuated SIV (52), these studies reinforce the significance of Nef-dependent demyelination observed in our research. The damaging impact of Nef on oligodendrocytes and myelin may contribute to the motor deficits and cognitive impairments that characterize HAND.

## Therapeutic implications

The reviewed studies suggest several potential therapeutic strategies for mitigating the neurotoxic effects of Nef and improving the management of HAND. Beyond directly targeting Nef with small-molecule inhibitors (53), blocking Nef's interference with cholesterol efflux presents a promising approach to protecting oligodendrocytes (19) and preserving myelin integrity. This can be achieved through inducers of expression of ABCA1, the main cellular effector of cholesterol efflux, by such agents as LXR agonists (54), or inhibitors that disrupt the interaction between Nef and calnexin, thereby preventing Nef-mediated impairment of ABCA1 maturation (55). Notably, this strategy may also help reduce Nef-driven inflammation (5).

Furthermore, the recent discovery that Nef is exposed on Nef EVs (56) opens the possibility of specifically targeting these pathogenic entities using monoclonal antibodies. Monoclonal antibody therapies have revolutionized the treatment of various diseases by providing precise and effective interventions. Examples include autoimmune diseases such as rheumatoid arthritis, treated with anti-TNF antibodies (57); infectious diseases like COVID-19, managed with SARS-CoV-2 spike protein-targeting antibodies (58); and cancers treated with anti-PD-1 antibodies (59). To apply this approach to neutralizing Nef EVs, it will be essential to identify a high-affinity antibody targeting a conserved region of Nef, ensuring broad efficacy against all Nef variants.

## Conclusions

The current studies contribute to the growing body of evidence supporting Nef's central role in the pathogenesis of HAND. Its involvement in neuroinflammation, oligodendrocyte damage, and myelin impairment highlights Nef as a promising therapeutic target in HIV-1 infection. The identification of extracellular vesicles carrying Nef as mediators of neuronal injury further reinforces the potential of targeting Nef EVs for intervention. A key novel insight from these studies is the demyelination effect of Nef EVs, offering a potential explanation for myelin impairment in HAND. Future research should focus on delineating the precise molecular pathways through which Nef exerts its neurotoxic effects, as well as exploring therapeutic strategies to block these mechanisms and prevent or reverse CNS damage. A critical priority will be distinguishing the respective contributions of HIV-infected brain cells and the Nef EVs they produce to neurocognitive impairment. Additionally, the mechanisms underlying Nef-mediated myelin damage remain to be fully elucidated. Advancing our understanding in these areas will be crucial for accelerating the development of effective treatments targeting Nef-driven neurotoxicity. Given the persistent challenges in managing HAND, targeted interventions that neutralize Nef's neurotoxic effects could provide a much-needed strategy to improve neurological outcomes in individuals living with HIV.
